# Comparison of proprietary and fine-tuned large language models for multi-label classification of billing codes from radiology reports

**DOI:** 10.1007/s00330-026-12445-3

**Published:** 2026-03-14

**Authors:** Kamyar Arzideh, Henning Schäfer, Ahmad Idrissi-Yaghir, Bahadir Eryilmaz, Sina Warmer, Eva Maria Hartmann, Katarzyna Borys, Cynthia Sabrina Schmidt, Johannes Haubold, Lale Umutlu, Michael Forsting, Felix Nensa, René Hosch

**Affiliations:** 1 Central IT Department, Data Integration Center, University Hospital Essen, Essen, Germany; 2 Institute for Artificial Intelligence in Medicine, University Hospital Essen, Essen, Germany; 3 Institute for Transfusion Medicine, University Hospital Essen, Essen, Germany; 4 Institute of Diagnostic and Interventional Radiology and Neuroradiology, University Hospital Essen, Essen, Germany; 5 Center of Sleep and Telemedicine, University Hospital Essen—Ruhrlandklinik, Essen, Germany

**Keywords:** Fine-tuning, Gebührenordnung für Ärzte (GOÄ), Large language models, Natural language processing, Radiology billing

## Abstract

**Objectives:**

While large language models (LLMs) have shown promise in medical text analysis, their application in automated medical billing code extraction remains underexplored, particularly for the German medical fee schedule system (GOÄ). Therefore, an LLM was fine-tuned to perform multi-label classification of GOÄ codes from radiology reports automatically, and its performance was compared with state-of-the-art commercial and open-source LLMs.

**Materials and methods:**

Following ethics committee approval, we analyzed 499,601 radiology reports from 124,497 patients, containing 1,799,971 manually identified GOÄ codes as ground truth. The MediPhi-Instruct 4B model was fine-tuned using five-fold cross-validation. Performance was evaluated on the hold-out test set and compared against GPT-5, GPT-4.1, GPT-oss, Kimi-K2, Deepseek-R1, Deepseek-V3, Gemini 2.5, Llama-70B, and Qwen-3 LLMs on a subset of 500 anonymized and 350 cleaned reports using zero-shot and few-shot prompting techniques.

**Results:**

The fine-tuned model achieved an accuracy of 77.15% ± 0.47% and a micro-average F1-score of 87.79% ± 0.31% on the hold-out test set. On a subset of 500 real-world samples, our models outperformed the best-performing LLM, Gemini 2.5 Flash, with an F1-score of 70.32% ± 1.54% compared to 58.22% ± 1.50% (*p* < 0.001). For the cleaned dataset of 350 samples, GPT-5 achieved the best F1-score of 89.51 ± 1.52% and outperformed the fine-tuned models (*p* < 0.001).

**Conclusions:**

Fine-tuned LLMs can effectively automate GOÄ code classification from radiology reports, with the potential of outperforming commercial LLMs. This approach shows promise for improving billing efficiency and accuracy in healthcare settings, though manual verification is still recommended.

**Key Points:**

***Question*** *LLMs with high parameters possess medical knowledge, but how effective are they at predicting billing codes from radiology reports compared to smaller, fine-tuned models*?

***Finidngs*** *A fine-tuned ensemble model achieved competitive results and can outperform larger, proprietary LLMs*.

***Clinical relevance*** *Smaller, fine-tuned models offer an efficient alternative to proprietary LLMs in generating billing codes and can be integrated to assist clinical coding. This technology has the potential to transform clinical billing procedures, but its use should be overseen by qualified professional personnel*.

**Graphical Abstract:**

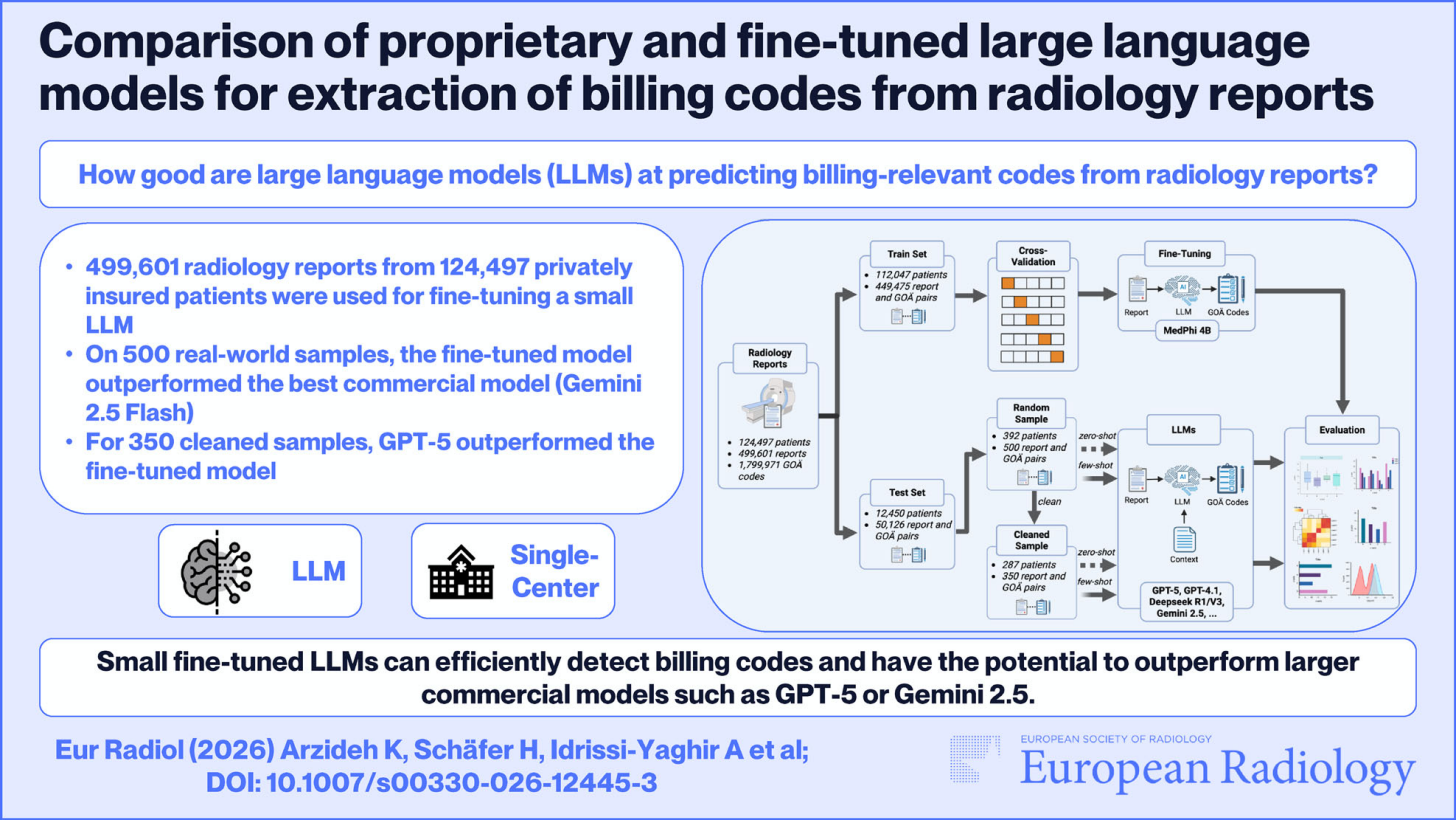

## Introduction

In modern healthcare systems, efficient and accurate billing practices are crucial for maintaining financial stability and ensuring proper reimbursement for medical services. One particular area that presents challenges is the billing of private medical services, which often requires careful review and coding of clinical documentation. In Germany, this process is governed by the “Gebührenordnung für Ärzte” (GOÄ), which is a fee schedule system for physicians, similar to Current Procedural Terminology (CPT) in the United States, that provides a standardized system for billing private medical services. Traditionally, the extraction of billing codes from clinical documents, such as radiology reports, has been a manual and time-consuming process [1]. Clinical controlling staff or other healthcare personnel must carefully review each report, identify relevant services that qualify as private, and assign the appropriate codes [2]. This labor-intensive task is not only time-consuming but also prone to human error, potentially leading to billing inaccuracies and revenue loss [3].

Recent advancements in artificial intelligence, particularly in the field of Natural Language Processing (NLP), have opened new avenues for automating complex text analysis tasks. Large Language Models (LLMs) have demonstrated remarkable capabilities in understanding [4, 5] and generating human-like text [6, 7] in the medical domain. In radiology, several studies have examined the potential of using LLMs [8–10]. However, the majority of publications focus their research on using proprietary LLMs with a high number of parameters, like ChatGPT [8, 9, 11–13]. Only a small number of articles used or fine-tuned comparatively small open-source LLMs for specific tasks [10, 14, 15]. Smaller LLMs are especially useful for narrow use-cases and can be included in agentic systems that consist of multiple expert models for specific tasks [16].

In this paper, we present an automated approach for classifying GOÄ codes from unstructured radiology reports using a fine-tuned 4-billion-parameter LLM. Utilizing routine hospital data, we fine-tuned an open-source model to automate this time-intensive task and benchmarked it against state-of-the-art commercial systems. Beyond predictive performance, this study contrasts two deployment paradigms: cloud-based proprietary models vs secure, local open-source solutions. By evaluating a compact model, we aim to determine if a privacy-compliant, cost-effective on-premises solution can achieve competitive accuracy for medical billing, thereby overcoming key GDPR and infrastructure barriers to clinical adoption.

## Methods

### Study design

**Fig. 1:**
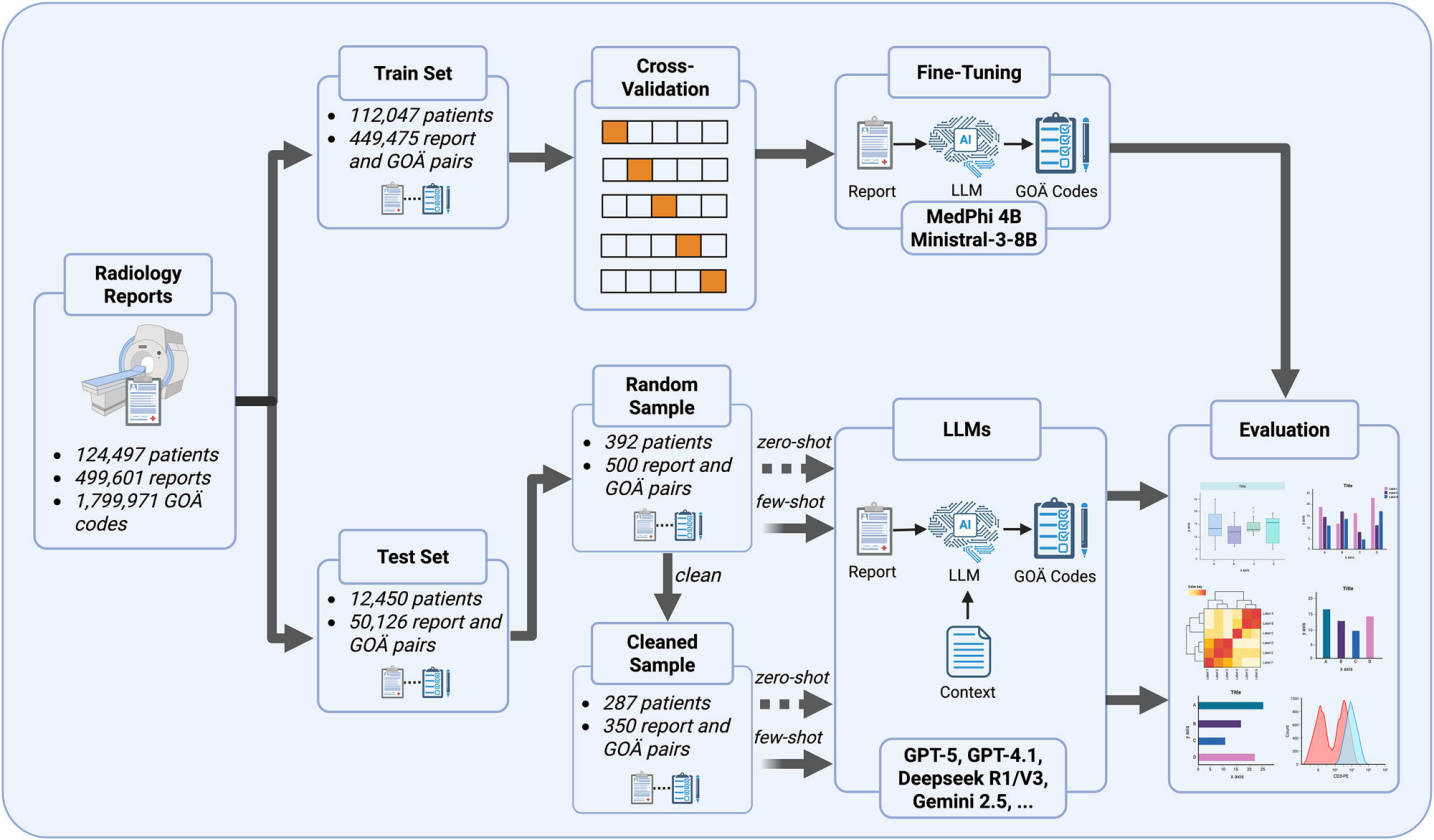
Methods and study design. The complete workflow of the study is summarized, including steps for dataset preparation, division into training and test set and fine-tuning. LLM, large language model; GOÄ, Gebührenordnung für Ärzte. The illustration was created with BioRender.com

This study was approved by the Ethics Committee of the Medical Faculty of the University of Duisburg-Essen (approval number 23-11557-BO, 20.02.2024). Due to the study’s retrospective nature, the requirement of written informed consent was waived by the Ethics Committee of the Medical Faculty of the University of Duisburg-Essen. This study adheres to the TRIPOD-LLM [17] guidelines where applicable. Figure 1 illustrates the dataset preparation steps and general workflow of the study.

### Inclusion and exclusion criteria

The study cohort included all radiology reports from privately insured adults (≥ 18 years) treated at the University Hospital Essen between 1999 and 2025 with documented GOÄ codes in the Picture Archive Communication System (PACS). No other demographic exclusions were made. For manual evaluation, 500 Real-World Samples were drawn from the validation set, excluding reports consisting solely of invariant normal-finding templates to ensure clinical diversity. From this subset, a cleaned sample (*n* = 350) was derived by excluding 150 reports dominated by institution-specific surcharge logic rather than extractable clinical text. Additionally, 163 individual codes (out of 586 total codes) representing pure administrative surcharges without textual correlations were removed from the ground truth to isolate semantic understanding.

### Dataset

A total of 499,601 radiology reports from 124,497 patients were retrieved using the Python programming language with pandas [18] (Version 2.3.1) and slqalchemy [19] package (Version 2.0.41). Demographic information of the patients was analyzed, and dataset characteristics like the number of tokens were calculated using the nltk [20] package (Version 3.9.1) and are presented in Table 1.

**Table 1:** Patient demographics and radiology report characteristics

	Radiology reports (*n* = 499,601)
Number of patients	124,497
Age (years), median (SD)	58 (SD: 21.93)
Sex (female), *n* (%)	57,441 (46.1%)
Total number of main diagnoses	270,670
Top 10 ICD-10 codes (main diagnosis)	I20.8 (angina pectoris) (*n* = 2712) (1%) C78.7 (liver or bile ducts cancer) (*n* = 2100) (0.78%) C73 (thyroid cancer) (*n* = 2029) (0.75%) C34.1 (lung cancer) (*n* = 1945) (0.72%) C34.9 (lung cancer: unspecified) (*n* = 1931) (0.71%) C61 (prostate cancer) (*n* = 1810) (0.67%) I25.13 (three-vessel disease) (*n* = 1758) (0.65%) I20.0 (angina pectoris: unstable) (*n* = 1728) (0.64%) C79.3 (brain cancer) (*n* = 1529) (0.56%) D38.1 (trachea, bronchus, and lungs: unknown formation) (*n* = 1414) (0.52%)
Report characteristics	Total number of tokens: 55,346,170
	Total number of unique tokens: 296,425
	Total number of sentences: 6,488,120
	Average tokens per document: 110.78
	Average sentence length: 8.53

The table shows different statistics of the documents used and patients included for training and evaluation in this study. “Total number of tokens” was calculated by counting the number of words in the documents, including punctuation. The lemma form of each token was created, and the unique number of lemmas was used as an approximation for the “total number of unique tokens”. “Total number of sentences” represents the count of all sentences per document. “Average tokens per document” was calculated by dividing the total number of tokens by the total number of documents. “Average sentence length” was calculated by dividing the number of tokens by the total number of sentences

The radiology reports were originally retrieved from the hospital’s PACS in base64 encoded format. The first author performed the necessary preprocessing, which was limited strictly to decoding these strings into plain text using the built-in Python library base64 (Version 3.13.5). No additional text normalization techniques, such as stemming, lemmatization, or stop-word removal, were applied.

### Ground truth

A total of 1,799,971 manually documented GOÄ codes were retrieved from the separate hospital billing system to serve as ground truth. Comprising 238 unique codes, these labels are generally robust, but they inherently reflect historical documentation biases and potential human errors that the model may reproduce. The dataset exhibits class imbalance (see Supplementary Fig. S1), where a small subset of high-frequency codes dominates the distribution.

### Data partitions

The dataset was split 90/10% into a training and a test set on a patient basis, so that a patient’s data was either part of the training or the test set. After the initial split, five-fold cross-validation was performed on the training data. Five models were trained for each cross-validation fold. The initial 10% hold-out test set was used for evaluation.

### Model training

The *MediPhi-Instruct* model from Microsoft was used as the basis for further fine-tuning and evaluation. This model has 4B parameters and has already been trained on clinical data and achieved state-of-the-art results on medical and clinical scenarios [21]. This model was further fine-tuned using pairs of radiology reports and GOÄ codes described in the previous section.

To benchmark domain adaptation against general capabilities, we fine-tuned Ministral-3-8B-Instruct (Mistral AI) as a state-of-the-art general-purpose baseline. The model was trained using identical hyperparameters and cross-validation splits to ensure strict comparability. All models were applied exclusively to German-language radiology reports in plain text format. No image data was processed. The input prompt, incorporating the radiology report as context, is detailed in Supplementary Fig. S2. Hyperparameters and Python packages used in the training phase are reported in Supplementary Note S1.

### Evaluation

To evaluate the performance of the model, machine learning metrics like precision (P), recall (R), and F1-score were computed. Additionally, accuracy was calculated as subset accuracy, where a prediction is considered correct only if the predicted set of GOÄ codes perfectly matches the ground truth set, without any false positives or false negatives. Additionally, given that this is a multi-label classification problem with an imbalanced label distribution, micro-average scores were calculated. While macro-averaging would provide equal weight to rare codes, which can be financially important, our primary objective was to evaluate the model’s potential to reduce the manual administrative workload. This workload is driven by high-frequency standard procedures. Therefore, micro-averaging was chosen to reflect the system’s effectiveness in automating the bulk of routine documentation, aiming to free up human expert time for the verification of complex, low-frequency cases. In the context of the evaluation task at hand, recall is considered to be the most important metric, given that potential financial losses may occur when the model fails to predict GOÄ codes that were relevant for billing.

#### Validation set

The dataset used for evaluation was the 10% hold-out test set (see *Data Partitions* section). The set consisted of 50,126 radiology reports and GOÄ code pairs. The fine-tuned models were evaluated against the total hold-out test set. For this, the five models were evaluated separately on the test set by building micro average scores. Additionally, the model results were combined using an ensemble method. Only if a code was predicted by at least three models was it considered an ensemble choice and compared against the ground truth. The results of the ensemble were calculated using micro-average scores for each report separately (instance-based).

#### Comparison with larger LLMs

To compare the trained models with state-of-the-art LLMs, 500 samples from the hold-out test set were drawn. More information about the selection is described in the Inclusion and Exclusion Criteria section. For data privacy reasons, the radiology reports were anonymized using a de-identification pipeline developed at the investigating site [22]. The reports were also manually checked, and all sensitive information contained in the reports was removed to comply with European General Data Protection Regulation laws. The predicted codes of the models were compared against the ground truth, and the performance of the fine-tuned models was evaluated using the ensemble technique.

The results of the fine-tuned ensemble models were compared against different state-of-the-art LLMs. The models that were used for comparison were GPT-5, GPT-4.1 [23], GPT-oss, kimi-K2, deepseek-R1 [24], deepseek-V3 [25], gemini-2.5-pro [26], gemini-2.5-flash [26], Llama-3.3-70B-Instruct [27], and Qwen3-235B-A22B-FP8 [28]. A comprehensive specification of all evaluated models, including their accessibility status (open source vs proprietary), manufacturer company, parameter counts, and access methods, is provided in Table 2. Other generation parameters like temperature, top-p, maximum tokens, and number of generations per case are displayed in Supplementary Table S1. Further information about the evaluated models is also provided in Supplementary Note S2.

**Table 2:** Information about evaluated models

Model name	Company	Weights	Parameter	Access
gpt-5	Open AI	Proprietary	Unknown	Paid API
gpt-4.1-2025-04-14	Open AI	Proprietary	Unknown	Paid API
gpt-oss-120b	Open AI	Open-source	120B	Local inference**
kimi-k2-0711-preview	Moonshot AI	Open-source	1 T (32B activated)	Paid API
deepseek-R1-0528	Deepseek AI	Open-source	685B	Paid API
deepseek-V3-0324	Deepseek AI	Open-source	685B	Paid API
gemini-2.5-pro	Google	proprietary	Unknown	Paid API
gemini-2.5-flash	Google	Proprietary	Unknown	Paid API
llama-3.3-70b-instruct	Meta	Open-source	70B	Local inference**
qwen3-235B-A22B-FP8	Qwen	Open-source	235B (22B activated)	Local inference**
mediPhi-instruct	Microsoft	Open-source	4B	Local fine-tuning
ministral-3-8B-instruct	Mistral AI	Open-source	8B	Local fine-tuning

These LLMs were evaluated for the extraction of billing codes from radiology reports. The parameter size of some models is still unknown to the public. “Paid API” indicates the model was accessed via the official provider’s API service, which incurred per-token costs

^**^ “Local Inference” indicates the models were deployed on-premise and accessed locally. Note: While Kimi and DeepSeek are open-weight models, they were accessed via their respective API endpoints for this study

Inference on all models was performed using the OpenAI Python library (Version 1.97). During inference of larger models (GPT, Deepseek, Kimi), occasional server timeouts occurred. Therefore, a retry mechanism was implemented by automatically re-attempting the request until it was successful and a valid response was returned.

#### Prompt engineering

Initial zero-shot experiments (Supplementary Fig. S3) yielded poor performance, necessitating a context-rich approach. Consequently, a few-shot prompt was developed (Supplementary Fig. S4) incorporating official GOÄ code definitions structured by section and two consistent examples of high-frequency examinations (standard chest and combined chest/rib X-rays). Prompts were iteratively refined by a senior medical data scientist in consultation with a senior medical controller with more than 15 years of professional experience documenting GOÄ codes from radiology reports to ensure clinical accuracy. The final output was strictly constrained to a comma-separated list of codes without reasoning steps to ensure consistent parsing across all models.

#### Report types overview

**Fig. 2:**
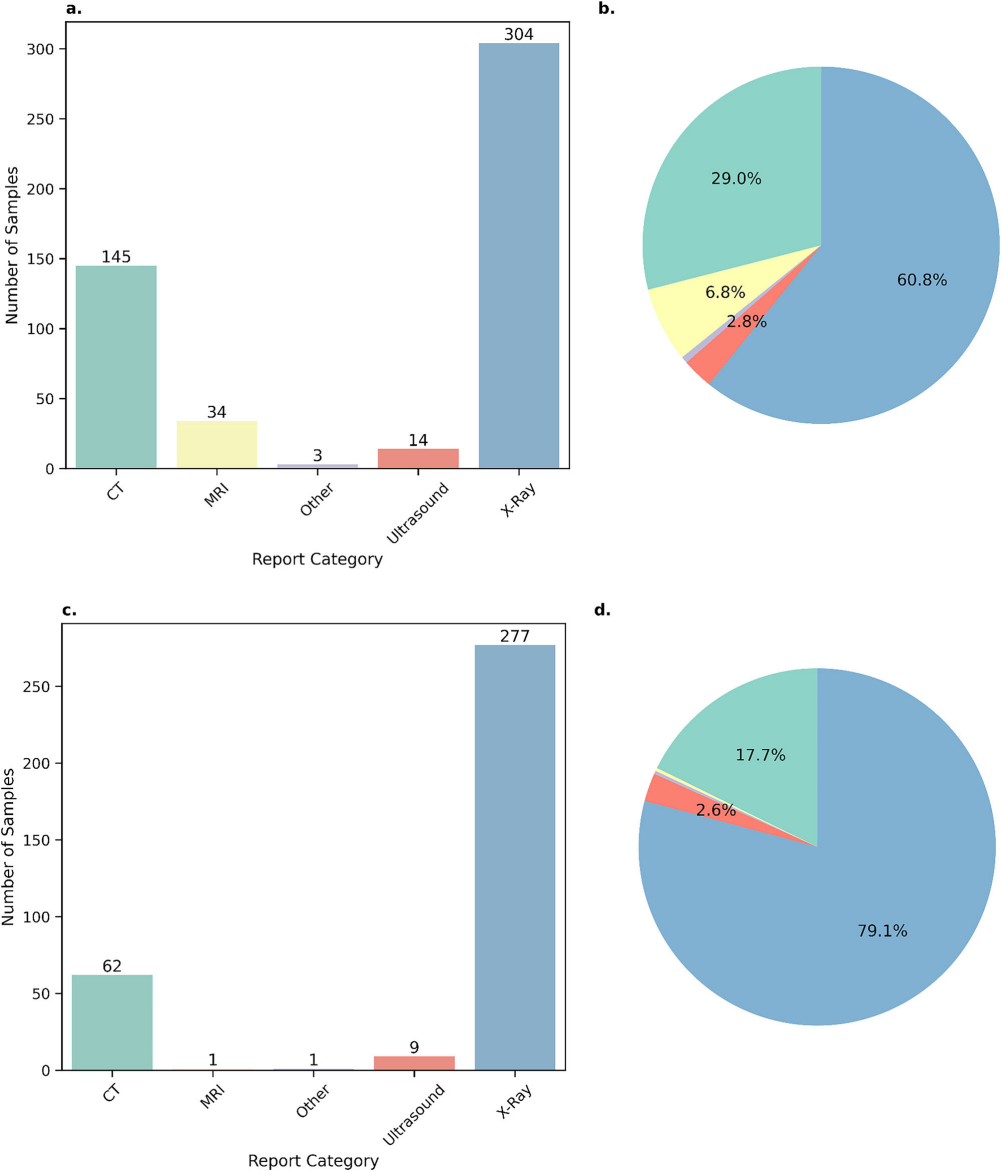
Frequency of imaging techniques described in the radiology report samples. **a** Real-world sample—report category frequency. **b** Real-world sample—distribution percentage. **c** Cleaned sample—report category frequency. **d** Cleaned sample—distribution percentage. The plots show the distribution of imaging procedures for the sample evaluation data

The sample dataset was categorized into different categories using the described imaging protocols mentioned in the reports. In Fig. 2, the distribution of imaging protocols present in the reports is visualized.

The most frequent imaging procedure was X-ray, followed by CT. For the real-world samples, MRI was the third most frequent procedure. Because the documentation of GOÄ codes for MRI reports often contains surcharge-based codes, most samples were filtered out for the second comparison.

### Statistical analysis

All variables and metrics in the manuscript were reported using mean and standard deviation (SD) if normally distributed and otherwise using median and interquartile range (IQR). The normality assumption was checked using the Shapiro–Wilk test [29]. In addition, 95% confidence intervals (95% CI) were reported based on cross-validation model performance metrics using 10.000 iterations of bootstrapping [30]. Performance metrics between the fine-tuned and best-performing zero-shot LLM were compared using a paired *t*-test [31]. All statistical tests were conducted using the Python package sklearn [32] (Version 1.7.0) and scipy [33] (Version 1.15.3).

## Results

### Performance on the validation dataset

The evaluation results of the fine-tuned model on the hold-out validation set are visualized in Supplementary Fig. S5. The five cross-validated models achieved an accuracy of 77.15% ± 0.47% (95% CI: 76.71%–77.52%). The micro-average F1-score was 87.79% ± 0.31% (95% CI: 87.52%–88.05%). As the precision and recall results indicate, the model shows a stronger precision performance. The mean precision is 91.11% ± 0.61% (95% CI: 90.51%–91.56%) compared to a mean recall of 84.71% ± 0.15% (95% CI: 84.56%–84.82%).

The ensemble model achieved an instance-based accuracy of 85.54% ± 0.28% (95% CI: 85.30%–85.79%) and a micro-average F1-score of 88.62% ± 0.24% (95% CI: 88.40%–88.84%). The ensemble model also performed higher precision with a micro-average score of 92.88% ± 0.22% (95% CI: 92.68%–93.07%) compared to a recall score of 88.68% ± 0.26% (95% CI: 88.44%–88.91%).

MediPhi-Instruct consistently outperformed the general-purpose Ministral baseline, achieving a superior mean micro-F1 of 87.79% ± 0.31% (vs 85.83% ± 0.25%) and a higher ensemble score (88.62% vs 87.64%). Detailed comparative metrics are provided in Supplementary Table S2.

### Performance comparison with LLMs

#### Real-world dataset

**Fig. 3:**
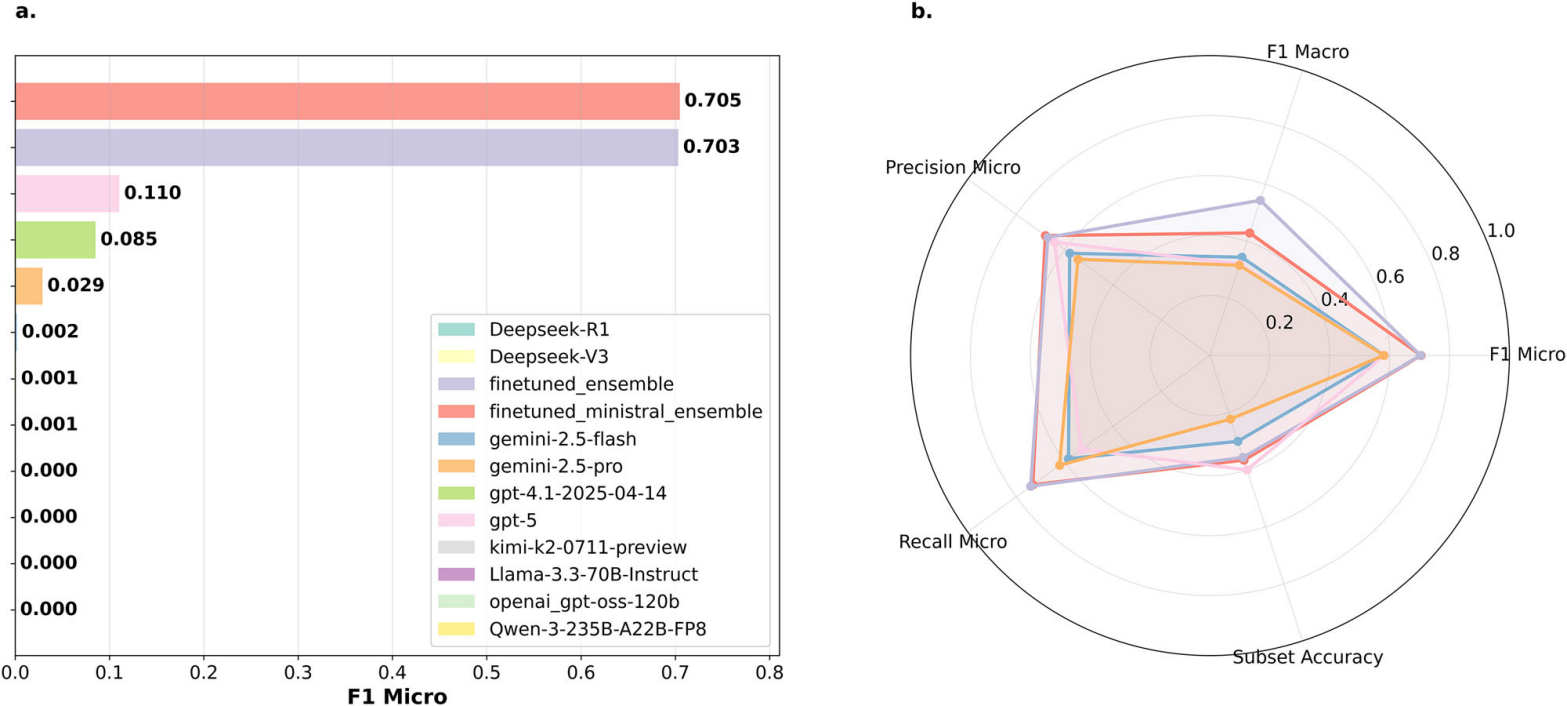
Comparison of LLMs and Fine-tuned Model on Real-World Sample. **a** Zero-shot performance. **b** Few-shot performance of the top five models. The dataset for this evaluation was created by taking a sample of 350 radiology reports from the holdout test set. The reports were anonymized and given as input to the different LLMs using zero-shot and few-shot prompting techniques. The results of the fine-tuned model were calculated by using an ensemble technique over the five fine-tuned models

The results of the comparison between the five fine-tuned models and different LLMs on 500 anonymized radiology reports are visualized in Fig. 3.

The fine-tuned ensemble model reached a micro-average F1-score of 70.32% ± 1.54% (95% CI: 67.33%–73.28%). In comparison, gpt-5 achieved a F1-score of 11.04% ± 1.17% (95% CI: 8.79%–13.38%) for the zero-shot (Figs. 3a) and 58.15% ± 1.58% (95% CI: 55.05%–61.23%) for the few-shot task (Fig. 3b). Gemini-2.5-flash achieved a slightly higher F1-score for the few-shot task of 58.22% ± 1.50% (95% CI: 55.26%–61.13%) while gemini-2.5-pro performed worse with a F1-score of 58.12% ± 1.42% (95% CI: 55.35%–60.88%). The highest Recall score of 74.05% ± 2.25% (95% CI: 69.47%–78.38%) was achieved by the fine-tuned ensemble model, whereas the best Recall for few-shot prompting was reached by gemini-2.5-pro with a score of 62.12% ± 2.09% (95% CI: 58.10%–66.27%). The comparison between the fine-tuned model and baseline Ministral-3-8B performance is provided in Supplementary Table S3, and the performances of each model are listed in Table 3.

**Table 3:** Comparison of LLMs and fine-tuned models for the extraction of GOÄ codes on real-world data

Model	Task	Precision	Recall	F1-micro	Accuracy
finetuned ministral-3 ensemble	Instruction-tuned	**0.680**	0.731	**0.705**	0.366
finetuned ensemble	Instruction-tuned	0.669	**0.740**	0.703	0.356
gemini-2.5-flash	Few shot	0.580	0.584	0.582	0.3
	Zero shot	0.001	0.126	0.002	0.0
gpt-5	Few shot	0.642	0.531	0.581	0.4
	Zero shot	0.126	0.098	0.110	0.01
gemini-2.5-pro	Few shot	0.545	0.621	0.581	0.22
	Zero shot	0.016	0.192		
Qwen3-235B-A22B-FP8	Few shot	0.565	0.468	0.512	0.292
	Zero shot	0.0	0.020	0.0	0.0
Deepseek-R1	Few shot	0.590	0.441	0.505	0.408
	Zero shot	0.0	0.0	0.0	0.0
gpt-4.1-2025-04-14	Few shott	0.579	0.432	0.495	**0.412**
	Zero sho	0.077	0.095	0.085	0.006
kimi-k2-0711-preview	Few shot	0.664	0.390	0.491	0.436
	Zero shot	0.0	0.001	0.001	0.0
Llama-3.3-70B-Instruct	Few shot	0.578	0.427	0.491	0.392
	Zero shot	0.0	0.002	0.0	0.0
Deepseek-V3	Few shot	0.528	0.430	0.474	0.37
	Zero shot	0.001	0.067	0.001	0.0
gpt-oss-120b	Few shot	0.556	0.379	0.451	0.378
	Zero shot	0.0	0.0	0.0	0.0

The table is sorted by performance with the best-performing model on top

Bold values represent the highest score in the respective metric and should help the reader to identify the bestperforming model

A *t*-test was performed by using the results of the best-performing LLM, gemini-2.5-flash, as a baseline and comparing its performance to the fine-tuned ensemble model. The results demonstrated statistically significant differences between the proposed model and baseline, with low *p*-values (*p* < 0.001) for accuracy, precision, recall, and F1-score. The notably high *t*-statistics indicate substantial performance gains, suggesting the proposed model offers a robust enhancement over the baseline approach.

#### Cleaned dataset

**Fig. 4:**
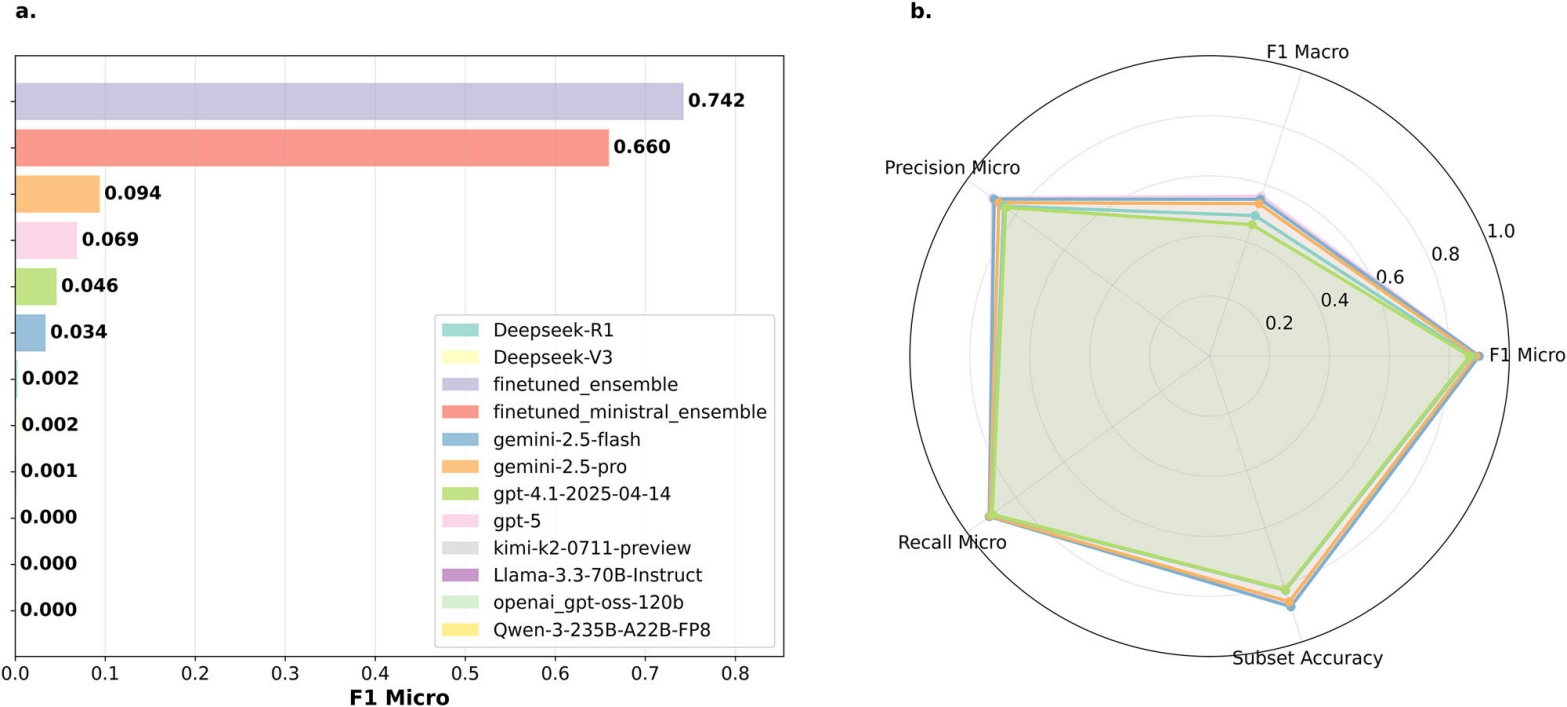
Comparison of LLMs and Fine-tuned Model on cleaned sample. **a** Zero-shot performance. **b** Few-shot performance of the top five models. The dataset for this evaluation was created by taking a sample of 350 radiology reports from the holdout test set. The reports were anonymized and given as input to the different LLMs using zero-shot and few-shot prompting techniques. The results of the fine-tuned model were calculated by using an ensemble technique over the five fine-tuned models

The LLM comparison results on the 350 cleaned samples are presented in Fig. 4.

GPT-5 achieved the best micro-average scores for the few-shot task with a F1-score of 89.51 ± 1.52% (95% CI: 86.42%–92.37%) (Fig. 4b) and the second-best F1-score for the zero-shot task (Fig. 4a) with a F1-score of 6.9 ± 1.37% (95% CI: 4.36%–9.67%). Gemini-2.5-pro showed the best F1-score of 9.4 ± 1.5% (95% CI: 6.53%–12.43%) for the zero-shot task. In comparison, the fine-tuned ensemble model performed worse with a micro-average F1-score of 74.23 ± 1.41% (95% CI: 71.49%–76.94%). The best Recall score of 90.9% ± 1.50% (95% CI: 87.93%–93.77%) was achieved by gemini-2.5-flash. Recall performance of the fine-tuned ensemble model was 86.84% ± 1.69% (95% CI: 83.54%–90.10%), but Precision showed a performance drop to 64.84 ± 1.57% (95% CI: 61.76%–67.98%). Comparison of performance between the fine-tuned and the baseline Ministral-3-8B model is provided in Supplementary Table S4, and the results of all models are listed in Table 4.

**Table 4:** Comparison of LLMs and fine-tuned models for the extraction of GOÄ codes on cleaned data

Model	Task	Precision	Recall	F1-micro	Accuracy
gpt-5	Few shot	**0.884**	0.906	**0.895**	0.874
	Zero shot	0.061	0.076	0.068	
gemini-2-5-flash	Few shot	0.878	**0.909**	0.893	**0.877**
	Zero shot	0.029	0.041	0.033	
gemini-2-5-pro	Few shot	0.854	0.906	0.880	0.860
	Zero shot	0.077	0.114	0.092	
Deepseek-R1	Few shot	0.847	0.899	0.872	0.820
	Zero shot	0.002	0.003	0.002	
gpt-4.1-2025-04-14	Few shot	0.841	0.896	0.868	0.817
	Zero shot	0.034	0.066	0.045	
Llama-3.3-70B-	Few shot	0.838	0.848	0.843	0.797
Instruct	Zero shot	0.001	0.005	0.001	
kimi-k2-0711-	Few shot	0.850	0.830	0.840	0.811
preview	Zero shot	0.0	0.0	0.0	
Qwen3-235B-A22B-FP8	Few shot	0.834	0.803	0.818	0.751
	Zero shot	0.0	0.028	0.0	
gpt-oss-120b	Few shot	0.781	0.838	0.808	0.740
	Zero shot	0.0	0.0	0.0	
Deepseek-V3	Few shot	0.655	0.890	0.753	0.529
	Zero shot	0.001	0.025	0.002	
Finetuned ensemble	Instruction-tuned	0.648	0.868	0.742	0.463
Finetuned ministral-3 ensemble	Instruction-tuned	0.536	0.858	0.660	0.217

The table is sorted by performance with the best-performing model on top

Bold values represent the highest score in the respective metric and should help the reader to identify the bestperforming model

A *t*-test was performed by using the results of the fine-tuned ensemble model as a baseline and comparing its results to the best-performing LLM, gpt-5. The results demonstrated statistically significant differences between the proposed model and baseline, with low *p*-values (*p* < 0.001) for accuracy, precision, recall, and F1-score. The notably high *t*-statistics indicate substantial performance gains, suggesting the proposed model offers a robust enhancement over the baseline approach.

#### Performance per category

**Fig. 5:**
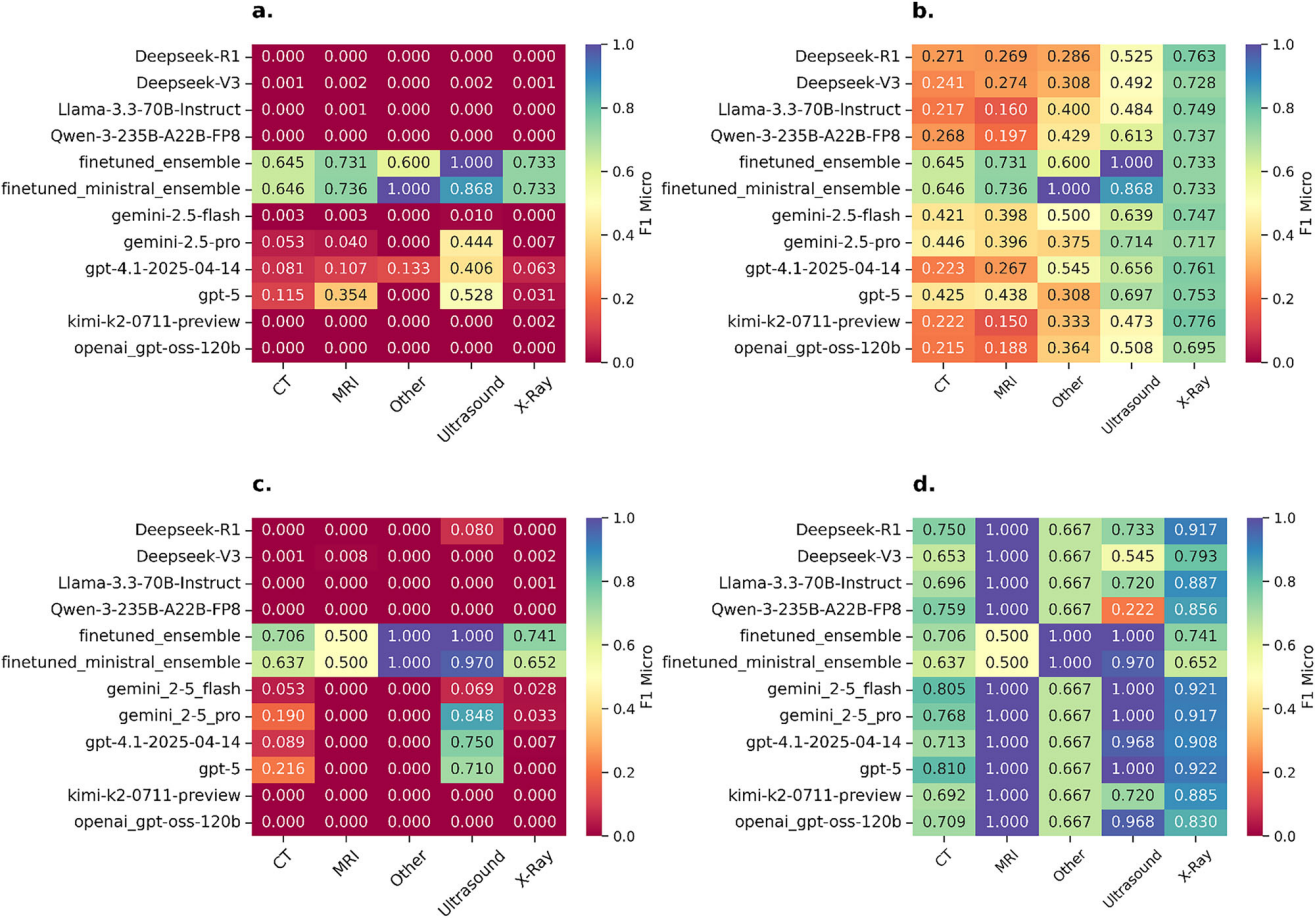
Model Performances per Imaging Technique. **a** Real-world sample—zero-shot performance. **b** Real-world sample—few-shot performance. **c** Cleaned sample—zero-shot performance. **d** Cleaned sample—few-shot performance. The results show differences in performance depending on the imaging technique

To provide a more detailed look at the performances of LLMs, the following section deals with the scores broken down by different imaging techniques that were documented in the radiology reports. The performance of the evaluated LLMs for each imaging technique is presented in Fig. 5.

For real-world samples, models generally showed low performance for zero-shot classification (Fig. 5a), with the fine-tuned model achieving the most consistent F1-scores, ranging from 60% (CT) to 100% (ultrasound). Other models struggled with the correct classification of GOÄ codes, especially for X-ray procedures with F1-scores between 0% (gpt-oss, gemini-2.5-flash, qwen3-235B-A22B-FP8, llama-3.3-70B, deepseek-R1) and 6.3% (gpt-4.1-2025-04-14). For the ultrasound category, the LLMs showed the best results with gpt-5 achieving an F1-score of 52.8% and Gemini-2.5-flash a F1-score of 44.4%. For the few-shot task (Fig. 5b), models showed the most consistent performances for X-ray procedures, with F1-scores varying from 69.5% (gpt-oss) to 77.6% (kimi-k2-0711-preview). For reports describing CT procedures, performances were more diverse with F1-scores between 21.5% (gpt-oss) and 64.5% (fine-tuned ensemble). In general, the models showed higher scores for X-ray and ultrasound procedures compared to CT and MRI for the few-shot task.

Similar to these results, the zero-shot performance on the cleaned sample dataset (Fig. 5c) also showed low F1-scores for the X-ray category, but better scores for ultrasound and CT. For ultrasound, the best performing model was the fine-tuned ensemble model (100%) followed by gemini-2.5-pro (84.8%) and gpt-4.1-2025-04-14 (75%).

The few-shot performances for the cleaned sample (Fig. 5d) also show high performances for X-ray and ultrasound procedures. Billing codes for MRI procedures are also recognized with high F1-scores. CT procedures in comparison showed lower F1-scores ranging from 65.3% (deepseek-V3) to 81% (gpt-5).

**Fig. 6:**
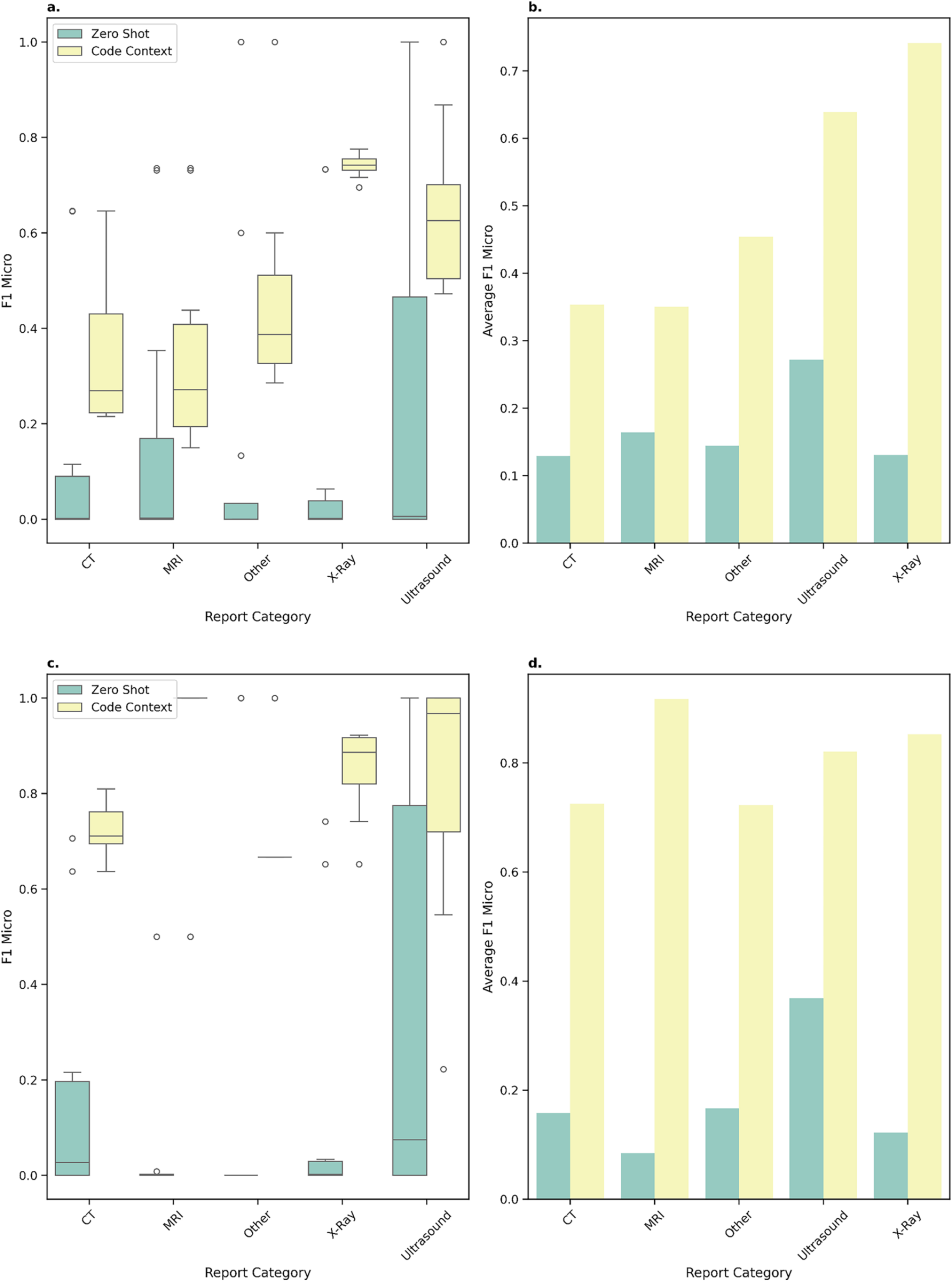
Average Model Performances per Imaging Technique. **a** Boxplots for the real-world samples—average F1-score of all models. **b** Barplots for the real-world sample—average F1-score of all models. **c** Boxplots for the cleaned sample—average F1-score of all models. **d** Barplots for the cleaned sample—average F1-score of all models. The average performance of all evaluated LLMs was calculated and broken down by imaging technique. The boxplots (**a**, **c**) show the performance range, while the barplots (**b**, **d**) show a comparison of the average performance

The average of model performances of each task and dataset was also calculated, and the results are presented in Fig. 6.

For zero-shot classification, codes for the categories ultrasound and CT were predicted with the best F1-scores (Fig. 6a, c). For the few-shot task, the X-ray category was predicted with high F1-scores for the real-world (Fig. 6b) and the cleaned sample dataset (Fig. 6d). The few-shot performances for the cleaned sample were more consistent across categories than for the real-world sample.

## Discussion

The results of this study indicate that the fine-tuned state-of-the-art LLMs are capable of predicting billing codes from unstructured reports. The fine-tuned LLM achieved overall competitive results, especially when considering the complex nature of billing regularities on the one hand and medical jargon on the other. Empirically, the smaller domain-adapted MediPhi-Instruct (4B) generally outperformed the larger general-purpose Ministral-3-8B, underscoring the critical value of domain-specific pre-training over pure parameter scaling in medical NLP. Future research should systematically benchmark a broader range of open-source architectures (e.g., 7B–70B) to define the optimal efficiency frontier between computational cost and downstream performance.

The performance gap between the full validation set (F1 88.6%) and the curated real-world sample (F1 70.3%) highlights the distinction between bulk automation and semantic generalization. The model excels at the high-frequency routine bulk that dominates clinical workflows. In the challenging real-world subset, the fine-tuned ensemble significantly outperformed the best few-shot model (Gemini-2.5-Flash, F1 58.2%; *p* < 0.001). Although GPT-5 achieved superior accuracy on the simplified Cleaned dataset (F1 89.5%), the fine-tuned model offers a more viable trade-off for deployment as it requires minimal inference context, incurs no per-token API costs, and operates efficiently on local hardware.

Comparisons between existing studies are difficult, because, to our knowledge, there are no prior studies that fine-tuned or evaluated LLMs on a GOÄ code classification task. However, some studies focus on the extraction of CPT codes by physicians or LLMs. Duszak et al [34] analyzed the appropriate use of CPT codes of two experienced physician coders. They found out that only 82% of encounters were initially coded correctly with a tendency towards undercoding. Direct comparisons with our study are not feasible due to the differences in the coding systems and evaluation methods, but there are indications that physicians also struggle with this complex and error-prone task. Zaidat et al [35] investigated the ability of ChatGPT to classify CPT codes from operative notes. In a set of 50 notes, ChatGPT extracted 22 different possible codes with an area under the precision-recall curve ranging from 0.63 to 0.76, depending on the trial. Shost et al [36] trained and evaluated an AI model to classify seven primary surgical operation codes across different surgical categories. Categories were classified with a weighted accuracy of 91%.

A decisive advantage of the fine-tuned approach is data sovereignty. Unlike proprietary cloud-based models that require external data transfer, the lightweight MediPhi-Instruct model can be deployed on-premises, ensuring sensitive patient data never leaves the hospital firewall in compliance with GDPR. Given the lower performance on rare codes, a human-in-the-loop workflow remains essential. By functioning as a pre-screening system that populates probable codes for verification, the model can significantly reduce administrative burdens and facilitate large-scale health economic auditing. Due to strict privacy regulations, the underlying datasets and model weights cannot be published, though the training code is made publicly available.

### Limitations

Our study is subject to several limitations. The reliance on single-center, retrospective data from a university hospital introduces institution-specific biases in documentation and billing practices, meaning the generalizability to other healthcare settings remains to be verified. Additionally, the ground truth was derived from historical administrative data that may contain propagated human errors rather than clinically validated gold-standard labels.

Methodologically, the necessary anonymization and exclusion of invariant templates altered the linguistic structure compared to raw clinical text. Furthermore, the systematic filtering applied to the Cleaned Sample, specifically the removal of reports dominated by institution-specific surcharge logic, shifted the data distribution. While necessary for a fair semantic comparison, this cleanup does not fully reflect the raw administrative complexity of the original workflow.

Finally, this study lacks external and prospective validation to assess performance stability over time. The deployment of LLMs also entails specific risks, including prompt sensitivity, the potential for hallucinations, and reliance on proprietary APIs where unannounced vendor updates could impact reproducibility.

## Conclusion

This study demonstrates that compact, fine-tuned LLMs can effectively automate complex medical billing tasks, achieving performance levels that potentially rival or surpass those of massive, general-purpose proprietary systems. Our findings challenge the assumption that increasing model scale is the only path to high performance in clinical NLP. Instead, they highlight the efficacy of domain-specific adaptation on high-quality institutional data. However, given the potential for residual errors in generative models, such tools should currently be implemented within a human-in-the-loop framework, serving to augment rather than replace expert medical controlling staff.

## Supplementary information

Electronic Supplementary Material

Supplementary Material

## Data Availability

The data that support the findings of this study are not openly available due to reasons of sensitivity. Requests regarding the dataset can be sent to datagovernance@uk-essen.de and will be reviewed. The underlying code for this study is made publicly available upon publication on GitHub and can be accessed via this link https://github.com/UMEssen/Radbill.

## Abbreviations

CPT: Current procedural terminology
GOÄ: German medical fee schedule system (Gebührenordnung für Ärzte)
LLM: Large language model
NLP: Natural Language Processing
