# Supplementary material for: Comparison of proprietary and fine-tuned large language models for multi-label classification of billing codes from radiology reports
Source: Eur Radiol. 2026 Mar 14;36(8):6218–31. doi: 10.1007/s00330-026-12445-3 (PMC13341730; doi:10.1007/s00330-026-12445-3)
Supplement: Supplementary file 1 — Electronic Supplementary Material [file 330_2026_12445_MOESM1_ESM.pdf]

Comparison of Proprietary and Fine-Tuned Large Language Models for Multi-Label Classification of Billing Codes from Radiology Reports

Electronic Supplementary Material

Supplementary Figure S1. Top 10 Most Frequent Codes.

The most frequent code 5298 stands for “supplement to the services according to numbers 5010 to 5290 when using digital radiography (image intensifier radiography)”.

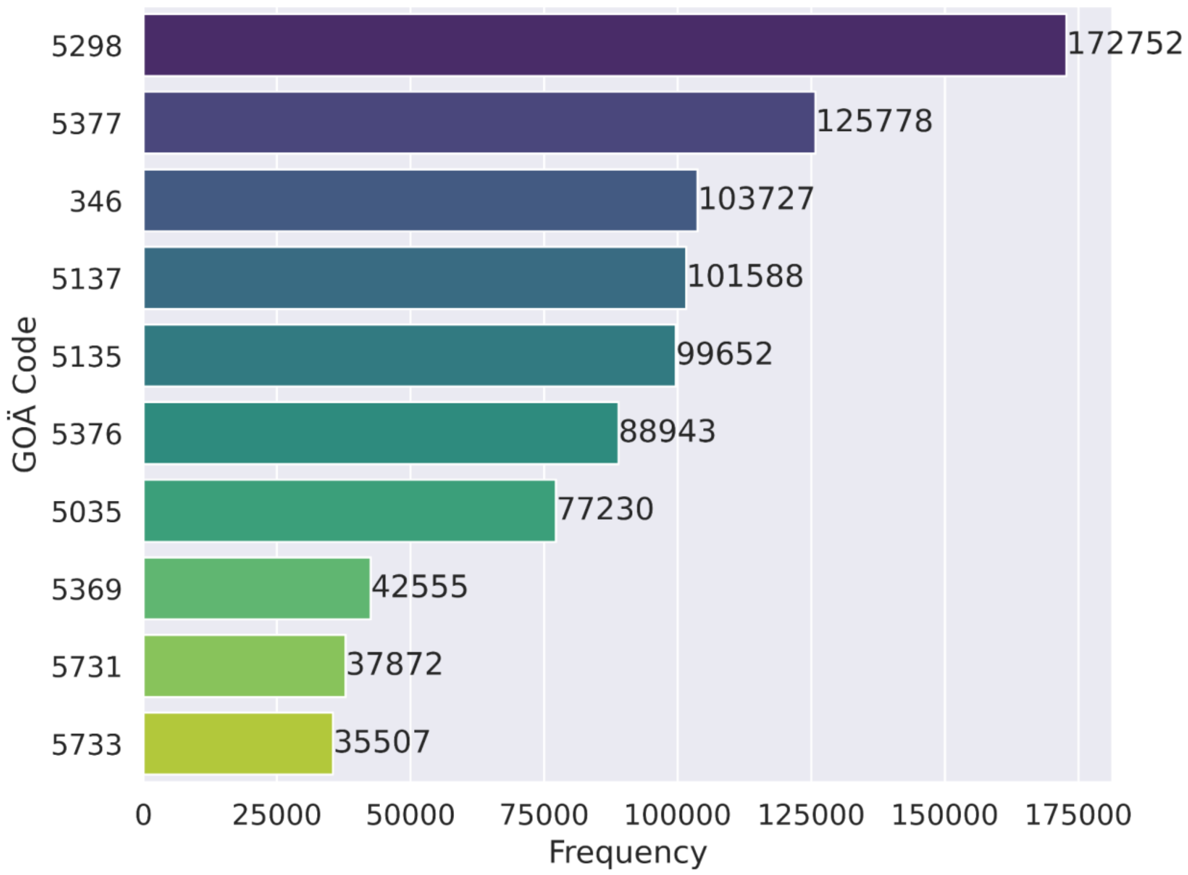

Supplementary Figure S2. Fine-tuning Prompt Example.

This prompt was used to fine-tune and evaluate a LLM for extracting GOÄ codes. The examples were originally written in German and translated to English. GOÄ stands for *Gebührenordnung für Ärzte* and is a fee schedule system for physicians.

**Instruction**	**Sample:**

```
### Task: You are a billing chatbot and your task is to extract codes from the fee schedule system for doctors (GOÄ codes) using radiology findings. Please identify the appropriate GOÄ codes from the following radiology report, separated by commas.

### Radiology Report:

Clinical data: Pre-operative visualisation of the lung
Question: effusion, infiltrate?

X-ray: Thorax in 2 planes from 19/07/2023:

Findings and assessment:
Initial examination (of this region); no previous external images were submitted.
Heart regular in shape, size and position.
Mediastinum and hilar structures unremarkable.
No signs of pulmonary congestion or inflammation.
No pneumothorax. No pleural effusion.
No evidence of intrathoracic tumour growth.

Age-appropriate cardiopulmonary findings.

### Answer:
5137, 5139
```

Supplementary Figure S3. Zero-Shot Prompt Example.

The prompt was used for the evaluation of proprietary models to classify GOÄ codes from radiology reports. The prompt was originally written in German and translated into English. GOÄ stands for *Gebührenordnung für Ärzte* and is a fee schedule system for physicians.

Instruction:

```
### Task: You are a billing chatbot and your task is to extract codes from the fee schedule system for doctors (GOÄ codes) using radiology findings. Please identify the appropriate GOÄ codes from the following radiology report, separated by commas. Please return only the GOÄ codes without any explanation or justification.
…

### Radiology Report:

Clinical data: Pre-operative visualisation of the lung
Question: effusion, infiltrate?

X-ray: Thorax in 2 planes from 19/07/2023:

Findings and assessment:
Initial examination (of this region); no previous external images were submitted.
Heart regular in shape, size and position.
Mediastinum and hilar structures unremarkable.
No signs of pulmonary congestion or inflammation.
No pneumothorax. No pleural effusion.
No evidence of intrathoracic tumour growth.

Age-appropriate cardiopulmonary findings.

### Answer:
5137, 5139
```

Supplementary Figure S4. Few-Shot Prompt Example.

The prompt was used for the evaluation of proprietary models to classify GOÄ codes from radiology reports. In this prompt, additional context about relevant GOÄ sections and two example reports/GOÄ pairs were provided. The prompt was originally written in German and translated into English. GOÄ stands for *Gebührenordnung für Ärzte* and is a fee schedule system for physicians.

Instruction:

Task: *You are a billing chatbot and your task is to extract codes from the German fee schedule system for doctors (GOÄ codes) using radiology findings.*

Please identify the appropriate GOÄ codes from the following radiology report, separated by commas. As this is a radiology finding, section O: ‘Radiological diagnostics, nuclear medicine, magnetic resonance imaging, and radiation therapy’ from the GOÄ classification is particularly relevant. (GOÄ sections 5000-5855).

However, sections B: 'Basic services and general services' (GOÄ sections 1-109) or C: ‘Non-area-related special services’ (GOÄ sections 200-449) may also be relevant.

Identify all services explicitly mentioned or implicitly described in the text that are billable according to GOÄ. Specify the corresponding GOÄ section for each service.

Guidance for selecting the correct GOÄ codes:

GOÄ Section B: Basic services and general services (codes 1-109):

- 2: Issuing repeat prescriptions and/or referrals and/or transmitting findings or medical orders – including by telephone – by the medical assistant and/or measuring physical conditions (e.g., blood pressure, temperature) without consultation, when the physician is consulted
- 3: In-depth consultation exceeding the usual scope – including by telephone

…

```
To help you extract the GOÄ codes, here are two examples of radiology findings and the corresponding GOÄ codes.

Example Radiology Report 1:
…

GOÄ Codes for Example 1: …


Example Radiology Report 2:

…

GOÄ Codes for Example 2:


### Radiology Report:

Clinical data: Pre-operative visualisation of the lung
Question: effusion, infiltrate?

X-ray: Thorax in 2 planes from 19/07/2023:

Findings and assessment:
Initial examination (of this region); no previous external images were submitted.
Heart regular in shape, size and position.
Mediastinum and hilar structures unremarkable.
No signs of pulmonary congestion or inflammation.
No pneumothorax. No pleural effusion.
No evidence of intrathoracic tumour growth.

Age-appropriate cardiopulmonary findings.
```

```
### Answer:
5137, 5139
```

Supplementary Figure S5. Evaluation Results of the Fine-tuned LLM.

The results of the five cross-validated models were calculated by micro averaging the scores over all five trained models. The ensemble model uses the five cross-validated models and decides if a code was predicted based on frequency of predictions. If a code is predicted by at least three models, it is considered a prediction of the ensemble model and compared against the reference standard.

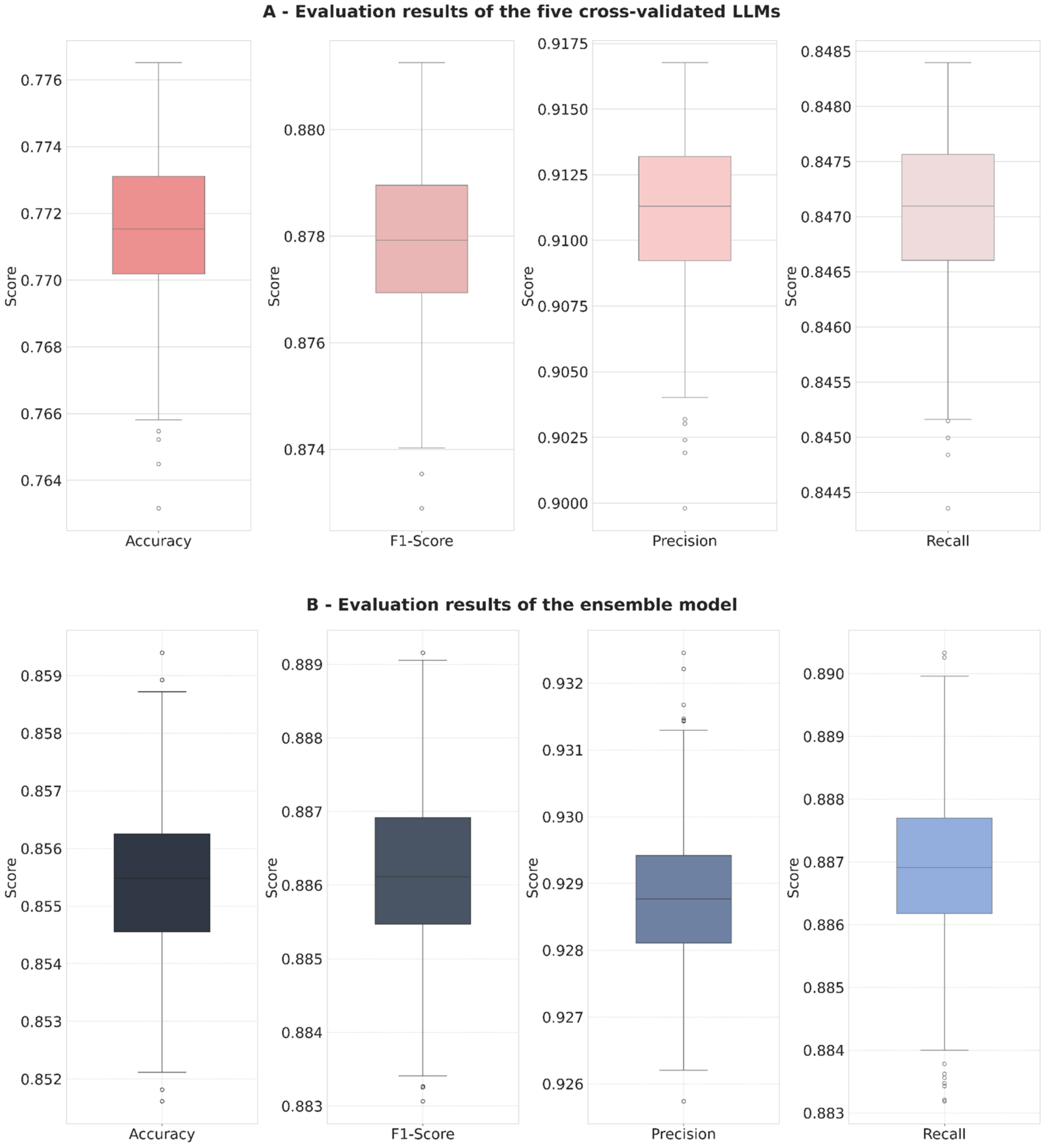

Supplementary Table S1. Generation Parameters per Model.

These are the generation parameters used for inference of the different models. Temperature was chosen based on the recommendations of the model creators. Otherwise, the default parameters of the *openai* library were used.

**Model Name**	**Temperature**	**Top-p**	**Maximum Tokens**	**Number of generations**
gpt-5	0	1	8000	1
gpt-4.1-2025-04-14	0	1	8000	1
gpt-oss-120b	0	1	8000	1
kimi-k2-0711-preview	0.6	1	8000	1
deepseek-R1-0528	0.6	1	8000	1
deepseek-V3-0324	0.6	1	8000	1
gemini-2.5-pro	0	1	8000	1
gemini-2.5-flash	0	1	8000	1
llama-3.3-70b-instruct	0	1	8000	1
qwen3-235B-A22B-FP8	0.6	1	8000	1
mediPhi-instruct	0	1	8000	1
ministral-3-8B	0	1	8000	1

Supplementary Table S2. Results of Fine-Tuned Models on Validation Set.

The following table presents the micro-averaged performance metrics on the validation set. The MediPhi-Instruct model is compared against the Ministral-3-8B-Instruct baseline. Results are grouped by "Average" (averaged across five-fold cross-validation) and "Ensemble" (majority voting). F1-scores for the Ensemble configuration were calculated as the average of instance-level F1-scores (per report) rather than the harmonic mean of the aggregated precision and recall. This accounts for variability at the individual report level.

Evaluation Method	Model	Accuracy	Precision	Recall	F1-Score
Average	MediPhi Instruct	**77.15% ± 4.7%**<br>[76.71–77.52]	**91.11% ± 6.1%**<br>[90.51–91.56]	**84.71% ± 1.5%**<br>[84.56–84.82]	**87.79% ± 3.1%**<br>[87.52–88.05]
Average	Ministral-3-8B-Instruct	75.18% ± 3.8%<br>[74.79–75.43]	90.81% ± 1.32%<br>[89.61–91.91]	81.38% ± 6.8%<br>[80.79–81.98]	85.83% ± 2.5%<br>[85.58–85.99]
Ensemble	MediPhi Instruct	**85.54% ± 2.8%**<br>[85.30–85.79]	**92.88% ± 2.2%**<br>[92.68–93.07]	**88.68% ± 2.6%**<br>[88.44–88.91]	**88.62% ± 2.4%**<br>[88.40–88.84]
Ensemble	Ministral-3-8B-Instruct	84.36% ± 2.9%<br>[84.11–84.62]	92.61% ± 2.3%<br>[92.41–92.81]	87.21% ± 2.7%<br>[86.97–87.45]	87.64% ± 2.5%<br>[87.41–87.87]

Supplementary Table S3. Results of Fine-Tuned Models on Real-World Sample.

The following table presents the micro-averaged performance metrics on the real-world evaluation sample. The MediPhi-Instruct model is compared against the Ministral-3-8B-Instruct baseline. Results are grouped by "Average" (averaged across five-fold cross-validation) and "Ensemble" (majority voting). F1-scores for the Ensemble configuration were calculated as the average of instance-level F1-scores (per report) rather than the harmonic mean of the aggregated precision and recall. This accounts for variability at the individual report level.

Evaluation Method	Model	Accuracy	Precision	Recall	F1-Score
Average	MediPhi Instruct	53.25% ± 1.2%<br>[52.17–54.32]	66.56% ± 0.8%<br>[65.88–67.24]	**72.67% ± 1.4%**<br>[71.48–73.86]	**69.48% ± 1.1%**<br>[68.56–70.4]
Average	Ministral-3-8B-Instruct	**52.83% ± 0.7%**<br>[52.27–53.39]	**66.99% ± 0.9%**<br>[66.16–67.73]	71.44% ± 1.1%<br>[70.44–72.31]	69.13% ± 0.6%<br>[68.65–69.61]
Ensemble	MediPhi Instruct	35.60% ± 2.1%<br>[31.40–39.80]	66.94% ± 1.7%<br>[63.25–70.25]	**74.05% ± 2.3%**<br>[69.47–78.38]	70.32% ± 1.5%<br>[67.33–73.28]
Ensemble	Ministral-3-8B-Instruct	**36.60% ± 2.1%**<br>[32.40–40.80]	**67.97% ± 1.7%**<br>[64.66–71.19]	73.15% ± 2.5%<br>[68.23–77.93]	**70.46% ± 1.6%**<br>[67.18–73.54]

Supplementary Table S4. Results of Fine-Tuned Models on Cleaned Sample.

The following table presents the micro-averaged performance metrics on the cleaned evaluation sample. The MediPhi-Instruct model is compared against the Ministral-3-8B-Instruct baseline. Results are grouped by "Average" (averaged across five-fold cross-validation) and "Ensemble" (majority voting). F1-scores for the Ensemble configuration were calculated as the average of instance-level F1-scores (per report) rather than the harmonic mean of the aggregated precision and recall. This accounts for variability at the individual report level.

**Evaluation Method**	**Model**	**Accuracy**	**Precision**	**Recall**	**F1-Score**
Average	MediPhi Instruct	**58.75% ± 0.6%** [58.16–59.22]	**64.55% ± 0.5%** [64.15–64.94]	**86.73% ± 0.6%** [86.23–87.24]	**74.01% ± 0.5%** [73.57–74.39]
Average	Ministral-3-8B-Instruct	56.58% ± 2.4% [54.46–58.53]	63.06% ± 1.9% [61.40–64.73]	84.56% ± 2.1% [82.60–86.08]	72.24% ± 1.9% [70.49–73.83]
Ensemble	MediPhi Instruct	**46.29% ± 2.7%** [41.14–51.71]	**64.84% ± 1.6%** [61.76–67.98]	**86.84% ± 1.7%** [83.54–90.10]	**74.24% ± 1.4%** [71.46–76.99]
Ensemble	Ministral-3-8B-Instruct	21.71% ± 2.2% [17.43–26.00]	53.55% ± 1.2% [51.32–55.83]	85.82% ± 2.1% [81.68–89.71]	61.15% ± 1.3% [63.45–68.41]

Supplementary Note S1. Training Hyperparameters and Python packages.

Training and inference were performed on a single Nvidia H100 GPU. For hyperparameters, a batch size of two and an initial learning rate of 0.0003 were employed. Training was limited to one epoch because preliminary experiments indicated that the model converged rapidly due to the large dataset size. Other parameters included a gradient accumulation step of four and the use of adafactor as optimizer. The packages involved in model training were transformers (Version 4.57.0), wandb (Version 0.21), datasets (Version 4.0.0), pytorch (Version 2.5.1), trl (Version 0.19.1) and sklearn (Version 1.7.0).

Supplementary Note S2. Details about the evaluated models.

GPT-5 is the newest version of models available from OpenAI and builds the next iteration of the GPT family after its predecessor gpt-4.1. In contrast to these proprietary models, gpt-oss is the first generative open-source model from Open AI since GPT-2. Deepseek-R1 is the reasoning model from Deepseek AI, and according to its authors, achieves performance comparable to gpt-4.1 across math, code, and reasoning tasks. Deepseek-V3 is a Mixture-of-Experts (MoE) chat model without reasoning capabilities that, according to its authors, was trained using an efficient and stable process. Gemini-2.5-pro and flash are both part of the gemini-2.5 family, which are the currently newest models from Google and available as stable versions. The flash models are more lightweight and faster; the pro models are more capable in most scenarios. Llama-3.3-70B-Instruct is an open-source model from Meta and, according to its authors, natively supports multilinguality, coding, reasoning, and tool usage. Qwen3-235B-A22B-FP8 is an 8-bit quantized MoE model with reasoning capabilities similar to the Deepseek-R1 model.
